# Gastrointestinal Goblet Cell Adenocarcinomas Harbor Distinctive Clinicopathological, Immune, and Genomic Landscape

**DOI:** 10.3389/fonc.2021.758643

**Published:** 2021-11-05

**Authors:** Dong-Liang Lin, Li-Li Wang, Peng Zhao, Wen-Wen Ran, Wei Wang, Long-Xiao Zhang, Ming Han, Hua Bao, Kaihua Liu, Xue Wu, Yang Shao, Xiao-Ming Xing

**Affiliations:** ^1^ Department of Pathology, The Affiliated Hospital of Qingdao University, Qingdao, China; ^2^ Geneseeq Research Institute, Nanjing Geneseeq Technology Inc., Nanjing, China; ^3^ School of Public Health, Nanjing Medical University, Nanjing, China

**Keywords:** goblet cell adenocarcinoma, colorectal cancer, pathology, differential gene expression, immune cell infiltration, immunohistochemistry

## Abstract

Goblet cell adenocarcinoma (GCA) is a rare amphicrine tumor and difficult to diagnose. GCA is traditionally found in the appendix, but extra-appendiceal GCA may be underestimated. Intestinal adenocarcinoma with signet ring cell component is also very rare, and some signet ring cell carcinomas are well cohesive, having some similar morphological features to GCAs. It is necessary to differentiate GCA from intestinal adenocarcinomas with cohesive signet ring cell component (IACSRCC). The goal of this study is to find occurrence of extra-appendiceal GCA and characterize the histological, immunohistochemical, transcriptional, and immune landscape of GCA. We collected 12 cases of GCAs and 10 IACSRCCs and reviewed the clinicopathologic characters of these cases. Immunohistochemical stains were performed with synaptophysin, chromogranin A, CD56, somatostatin receptor (SSTR) 2, and Ki-67. Whole transcriptome RNA-sequencing was performed, and data were used to analyze differential gene expression and predict immune cell infiltration levels in GCA and IACSRCC. RNA-sequencing data for colorectal adenocarcinoma were gathered from TCGA data portal. Of the 12 patients with GCA, there were 4 women and 8 men. There were three appendiceal cases and nine extra-appendiceal cases. GCAs were immunohistochemically different from IACSRCC. GCA also had different levels of B-cell and CD8+ T-cell infiltration compared to both colorectal adenocarcinoma and cohesive IACSRCCs. Differential gene expression analysis showed distinct gene expression patterns in GCA compared to colorectal adenocarcinoma, with a number of cancer-related differentially expressed genes, including upregulation of TMEM14A, GOLT1A, DSCC1, and HSD17B8, and downregulation of KCNQ1OT1 and MXRA5. GCA also had several differentially expressed genes compared to IACSRCCs, including upregulation of PRSS21, EPPIN, RPRM, TNFRSF12A, and BZRAP1, and downregulation of HIST1H2BE, TCN1, AC069363.1, RP11-538I12.2, and REG4. In summary, the number of extra-appendiceal GCA was underestimated in Chinese patients. GCA can be seen as a distinct morphological, immunohistochemical, transcriptomic, and immunological entity. The classic low-grade component of GCA and the immunoreactivity for neuroendocrine markers are the key points to diagnosing GCA.

## Introduction

Goblet cell adenocarcinoma (GCA) is a very rare tumor, formerly known as goblet cell carcinoid (1). GCA is composed of cells with secretory phenotypes, including goblet cells, endocrine cells, and Paneth cells. Whether these tumors are more closely related to neuroendocrine tumors or adenocarcinomas is controversial (2). Because GCAs are predominantly tumors of mucin secreting cells, they were reclassified as goblet cell adenocarcinomas in the current World Health Organization (WHO) classification of the digestive system (1). GCA was almost exclusively found in the appendix (1); however, more and more extra-appendiceal GCAs are being reported in the literature (3–6). We suspect that the number of extra-appendiceal GCA is underestimated, because GCA was considered as a special tumor that exclusively existed in the appendix. One of the objectives of our study is to look for extra-appendiceal GCAs.

Intestinal adenocarcinoma with signet ring cell component is also very rare in the intestinal tract, consisting of 0.1%–2.6% of colorectal cancer patients (7, 8). As we know, signet ring cell carcinoma usually is poorly cohesive (1). However, some signet ring cell carcinomas are well cohesive, having some similar morphological features to GCAs. We attempt to reveal the difference between GCAs and intestinal adenocarcinoma with cohesive signet ring cell component (IACSRCC). Early GCA mutational profiling showed it to be distinct from intestinal neuroendocrine tumors (NETs) and conventional intestinal adenocarcinoma (3, 9), where common genetic mutations in KRAS, GNAS, and APC were uncommon in GCAs (3, 9). However, no studies have to date investigated distinctions between GCA and colorectal adenocarcinoma and the morphologically similar IACSRCCs, in terms of both gene expression and the tumor immune microenvironment. Another aim of this study is to investigate whether GCA is a distinct entity in terms of transcriptional and immune landscape, which may facilitate an accurate diagnosis of GCA.

## Materials and Methods

### Case Selection

All GCAs, IACSRCC, and gastric carcinomas with signet ring cell components were reviewed to search for GCA and IACSRCC. To be classified as a GCA, the tumor must demonstrate at least a component of classic low-grade GCA (the classic low-grade tumor grows as tubules composed of goblet-like mucinous, variable numbers of endocrine cells, and Paneth-like cells with granular eosinophilic cytoplasm). The IACSRCC was defined as adenocarcinoma with a well-cohesive signet ring cell component rather than a poor-cohesive signet ring cell component. The diagnosis was confirmed by two pathologists (D-LL and L-LW). According to the morphology criteria, a total of 12 GCAs and 10 IACSRCCs were included in this study dating from 2016 to 2019. Out of the nine extra-appendiceal cases, six were initially misdiagnosed as signet ring cell carcinomas. All patients were from the Affiliated Hospital of Qingdao University. All clinicopathologic records and formalin-fixed paraffin-embedded (FFPE) sections were reviewed, and the results are summarized in **Table 1**. This study was performed according to the Declaration of Helsinki and was approved by the Ethics Committee of the Affiliated Hospital of Qingdao University on October 3, 2019 (No. QYFY WZLL 26478). All patients provided written informed consent.

**Table 1:** Clinicopathologic characteristics of GCAs.

Case No.	Age (years)/Sex	Location	Size (cm)	Histologic Findings				TNM stage	Follow-up (months)
				Grading	Non-GCA component	Vascular invasion	Perineural invasion		
1	70/M	Transverse colon	4.2	G3	Conventional adenocarcinoma	-	+	T3N0M0	DOD (45)
2	72/M	Appendix	4.3	G2	None	+	+	T3N2aM0	DOD (20)
3	57/M	Ascending colon	4.5	G2	None	+	-	T3N1bM0	DOD (5)
4	31/F	Sigmoid colon	3.5	G2	Conventional adenocarcinoma	+	+	T3N2aM0	DOD (18)
5	60/M	Rectum	6.5	G3	None	+	+	T4N2bM0	DOD (28)
6	36/F	Transverse colon	5.1	G3	Conventional adenocarcinoma	-	-	T3N1aM0	NET (38)
7	78/M	Stomach	4.9	G1	None	+	+	T4aN2M0	NET (28)
8	50/F	Stomach	5.0	G3	Large cell neuroendocrine carcinoma and conventional adenocarcinoma	+	+	T3N2M0	NET (28)
9	66/F	Sigmoid colon	4.0	G3	Mucinous carcinoma	-	+	T3N2bM0	NET (34)
10	65/M	Appendix	2.1	G2	None	+	+	T3N1M0	NET (25)
11	71/M	Stomach	3.5	G3	None	+	+	T3N1M0	NET (10)
12	67/M	Appendix	4.0	G1	None	+	-	T3N1M0	NET (18)

F, female; M, male; NET, no evidence of tumor; DOD, died of disease.

### TCGA-COAD Data

TCGA RNAseq gene expression data were obtained from NIH GDC data portal (https://portal.gdc.cancer.gov/). A total of 513 colorectal adenocarcinoma cases were selected for analysis.

### Immunohistochemistry

Immunohistochemical analysis was performed on paraffin-embedded sections using the following primary antibodies: synaptophysin (Clone: UMAB 112, Zhongshan, China), chromogranin A (Clone: LK2H10, Zhongshan, China), CD56 (Clone: 123C3, Roche, Switzerland), somatostatin receptor (SSTR)2 (Clone: EP149, Zhongshan, China), and Ki-67 (MIB1, Dako Cytomation, Denmark). All markers were performed using a VENTANA Benchmark ^®^ XT automated system (Ventana Medical Systems, Inc., Tucson, AZ, USA).

### RNA Extraction, Library Construction, and Whole Transcriptome Sequencing

Total RNA from FFPE samples were extracted using a RNeasy FFPE Kit (Qiagen). RNA purity was checked using Nanodrop 2000 for A260/280 and A260/A230 ratios (Thermo Fisher Scientific). All RNA samples were quantified by Qubit 3.0 using the RNA BR Assay Kit (Life Technologies). RNA integrity was assessed using Bioanalyzer 2100 (Agilent). RIN value (RNA integrity number) higher than 6.5 was required. RNA sequencing libraries were prepared using KAPA Stranded RNA-Seq Kit with RiboErase (KAPA Biosystems). Briefly, rRNA was first depleted with RiboErase, followed by DNase digestion for DNA removal. Purified RNA was subjected to first-strand cDNA synthesis, followed by second-strand synthesis with dUTP marking for strand specificity. This is followed by A-tailing, adapter ligation, and library amplification. Final library was quantified using KAPA Library Quantification Kit (KAPA Biosystems), and its fragment size distribution was analyzed using a Bioanalyzer 2100 (Agilent). Sequencing was performed on Illumina HiSeq4000 platform using PE150 sequencing chemistry (Illumina, USA) to an average of 60M reads per sample.

### RNA Sequencing Data Processing, Transcript Quantification, and Differential Gene Expression Analysis

FASTQ file quality control was performed using Trimmomatic (10), where N bases and low-quality (score <15) bases were removed. Reads aligning to rRNA and tRNA sequences were removed. Cleaned reads were aligned to the human reference genome (hg19) using STAR v 2.5.2b (11), a splice aware aligner. Transcripts were quantified using RSEM (12), which uses expectation maximization algorithm to optimally assign reads that maps to multiple transcripts. Differential gene expression analysis was performed using DESeq2 (13) R package.

### Pathway Enrichment Analysis

Gene ontology enrichment analysis (GOEA) was performed with GOATOOLS (14). Briefly, given a set of genes up- or downregulated in a certain group compared to another group, enrichment analysis will find gene ontology terms where these genes are overrepresented. Pathway and disease enrichment was performed using KOBAS 2.0 (15). Briefly, given a set of genes, KOBAS performs a statistical test to find pathways or disease in which the set of genes are overrepresented. KOBAS 2.0 uses five pathway databases (KEGG PATHWAY, PID, BioCyc, Reactome, and Panther) and five human disease databases (OMIM, KEGG DISEASE, FunDO, GAD, and NHGRI GWAS Catalog). Gene ontology terms represent biological function categories while pathway analysis involves enrichment for metabolic and signaling pathways.

### Immune Cell Infiltration Estimate

Relative infiltration level of six types of immune cells was estimated for each sample with TIMER (16) and RNAseq gene expression data. Six types of immune cells include CD4+ T cells, CD8+ T cells, B cells, neutrophils, macrophages, and myeloid dendritic cells.

### Immunomodulator Gene Expression Analysis

Level of immunomodulation was assessed as per previous study by Thorssen et al. (17). Gene expression levels in transcript per million (TPM) of 74 immunomodulator genes were assessed. These genes were divided into seven super categories, including receptor, ligand, co-stimulator, co-inhibitor, cell adhesion, antigen presentation, and other. They can also be immune checkpoint inhibitors, stimulators, or neither. Results were presented in a heatmap, where the median TPM of each gene was calculated for D: GCA and L: IACSRCC group, and *z*-score normalized across the two groups for each gene.

### Cytolytic Activity

Cytolytic activity was calculated as the geometric mean of gene expression levels of GZMA and PRF1 as previously described (18).

### Statistical Analysis

Non-parametric Wilcoxon’s rank-sum test was used to assess the differences between groups. *p* < 0.05 was considered to indicate a statistically significant difference. FDR was used for multiple testing correction. All statistical analyses were performed in R (v.3.5.3).

## Results

### Clinicopathological Characteristics

The clinicopathological characteristics of the patient of GCA are summarized in **Table 1**. The median age of the patients was 65.5 years (range 31–78 years). Of the 12 patients with GCA, there were 4 women and 8 men. The detailed locations were the appendix (three cases), the stomach (three cases), the transverse colon (two cases), the sigmoid colon (two cases), the ascending colon (one case), and the rectum (one case). The median tumor size was 4.0 cm (range 3.5–6.5 cm). The mean follow-up time was 28 months, ranging from 5 months to 46 months. Five patients died of cancer. Neither local recurrence nor distant metastasis had been found in the other seven patients. The clinicopathological characteristics of the patients with IACSRCC are summarized in **Table S1**.

### Histologic and Immunohistochemical Findings of GCAs and IACSRCC

**Figure 1:**
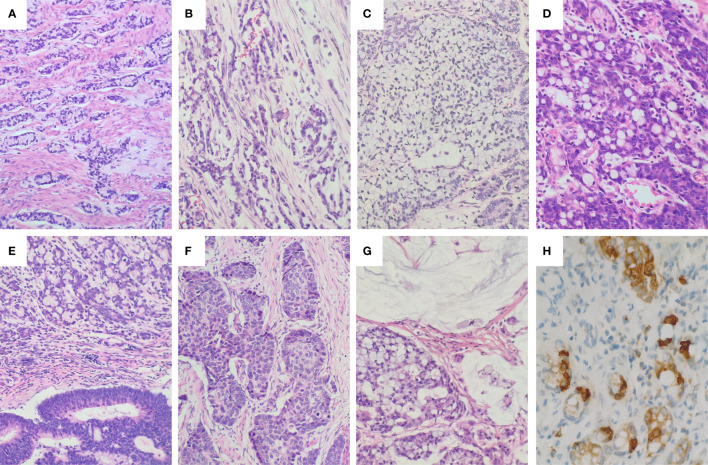
Histology and immunohistochemical staining of GCA. **(A)** The low-grade component of GCA showed round to oval discrete tumor clusters with or without lumens and simple trabecular growth consistent with tubules sectioned. **(B)** Single file growth lacking the clustered tubular architecture was a common representation of the high-grade component. **(C)** Very large aggregates of goblet cells in the high-grade component. **(D)** Fusion of goblet cell clusters to form anastomosing complex growth of goblet cell clusters in the high-grade components. **(E)** Conventional adenocarcinoma component in GCA. **(F)** Large cell neuroendocrine carcinoma component in GCA. **(G)** Mucinous carcinoma component in GCA. **(H)** The tumor cells were positive for Chromogranin **(A)** The tumor cells showed heterogeneous immunopositivity for Chromogranin A (Chromogranin A showed strong positivity in endocrine cells, but was negative or weakly positive in goblet cells and Paneth cells).

According to the latest WHO grading system (1), the 12 cases of GCAs were subdivided into Grade 1 (two cases), Grade 2 (four cases), and Grade 3 (six cases), respectively. The proportion of classic low-grade GCA components ranged from 5% to 90%. The classic low-grade GCA components grew as small tubular or clustered tumor clusters composed of goblet cells, in combination with cuboidal glandular cells and a variable number of Paneth-like cells (**Figure 1A**). The tumor cells in these clusters had a low N:C ratio, mild to at most moderate cytologic atypia, and infrequent mitoses (**Figure 1A**). The high-grade histologic components showed growth patterns of single file (**Figure 1B**), large aggregates (**Figure 1C**), and fused goblet cell clusters (**Figure 1D**). Non-GCA components existed in five cases, including conventional adenocarcinoma (**Figure 1E**), large cell neuroendocrine carcinoma (**Figure 1F**), and mucinous carcinoma (**Figure 1G**). Vascular invasion was positive in nine cases, and perineural invasion was detected in nine cases. The GCA component in all 12 cases stained positively for at least one of three neuroendocrine markers (synaptophysin, chromogranin A, and CD56). Chromogranin A was positive in 10 cases (83.3%) (**Figure 1H**), synaptophysin was positive in four cases (33.3%), and CD56 was positive in four cases (33.3%). SSTR2 was negative in all 12 cases. The Ki67 proliferative index ranged from 10% to 70% (see **Supplemental Table S2** for Ki67 index for each case).

**Figure 2:**
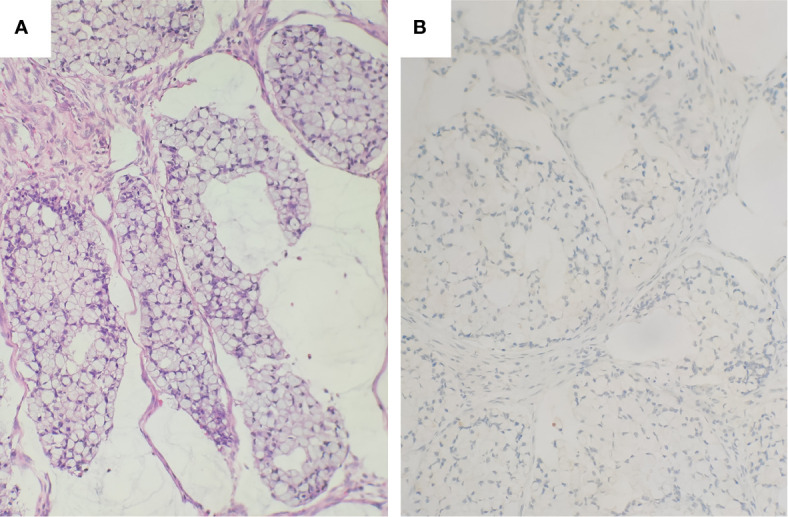
Histology and immunohistochemical staining of IACSRCCs. **(A)** The cohesive signet ring cell component in the IACSRCCs showed large well-cohesive signet ring cell aggregates rather than poor-cohesive signet ring cells, which was very similar to the large aggregates in the GCA. **(B)** The cohesive signet ring cell component in the IACSRCCs was negative for Chromogranin A.

The IACSRCCs were composed of large cohesive signet ring cell aggregates, resembling the large aggregates or fused goblet cell clusters in the high-grade component of GCAs (**Figure 2A**). The neuroendocrine markers were negative in 10 IACSRCCs (**Figure 2B**); only one case was focally positive for CD56, which was different from GCAs (*p* < 0.005). The immunohistochemical findings of GCAs and IACSRCCs are summarized in **Table 2** (for immunohistochemical findings at individual case level, see **Supplemental Table S2**).

**Table 2:** Summary of immunohistochemical findings of GCA and IACSRCC.

Antibody	GCA (+/total)	IACSRCC (+/total)	*p*-value
Neuroendocrine markers	12/12	1/10	0.000
Synaptophysin	4/12	0/10	
Chromogranin A	10/12	0/10	
CD56	4/12	1/10	
SSTR2	0/12	0/12	NA
Ki67 index	10%–70%	50%–90%	NA

The detailed immunohistochemical findings of GCAs are listed in **Supplementary Table S2**.

### GCA Has Distinct Immune Landscape Compared to Both Colorectal Adenocarcinoma and IACSRCC

**Figure 3:**
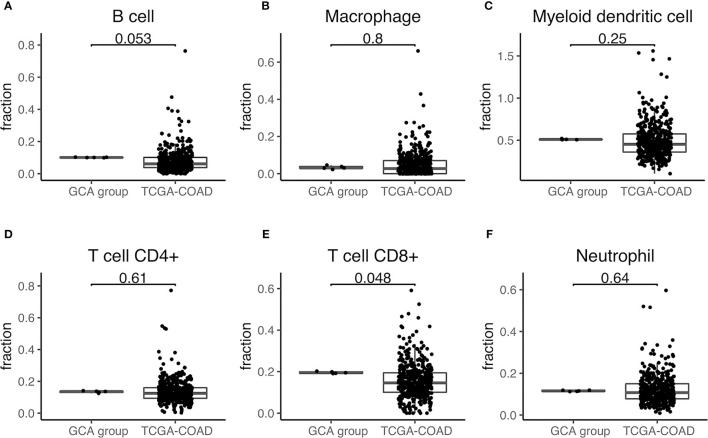
Immune cell infiltration of GCA *vs*. colorectal adenocarcinoma. **(A–F)** Immune cell infiltration estimate comparison. Infiltration % of six types of immune cells was estimated for each sample with TIMER using gene expression data from RNA-seq. Two-sided Wilcoxon test was used. *p*-value < 0.05 was seen as statistically significant. GCA, goblet cell adenocarcinoma; COAD, colorectal adenocarcinoma.

**Figure 4:**
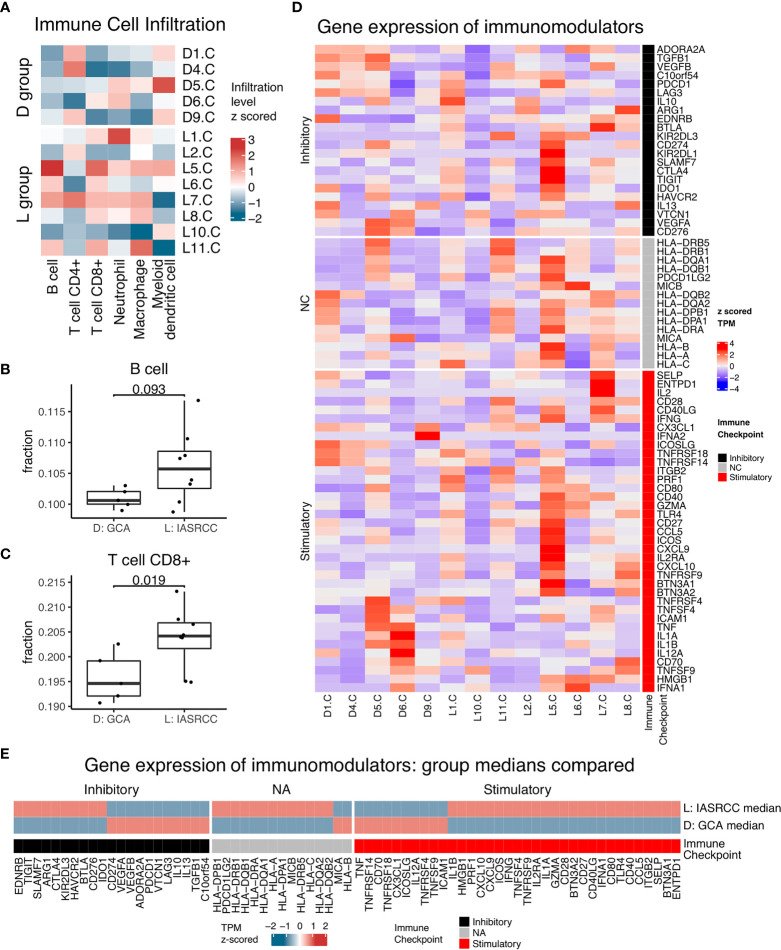
Immune landscape of GCA *vs*. IACSRCCs. **(A)** Immune cell infiltration estimate heatmap for samples in D: GCA and L: IACSRCC groups. Infiltration % of six types of immune cells was estimated for each sample with TIMER using gene expression data from RNA-seq. Infiltration level was *z*-scored across samples for each immune cell type. **(B, C)** Immune cell infiltration estimate comparison for D: GCA *vs*. L: IACSRCC group. Two-sided Wilcoxon test was used. *p*-value < 0.05 was seen as statistically significant. (Only significant or trend toward significance results are shown.) **(D)** Immunomodulators gene expression for samples in D: GCA and L: IACSRCC groups. Immunomodulation is characterized using gene expression level of 70 genes involved in immunomodulation (Thorsson, 2018). Gene expression levels were TPM (transcript per million) values *z*-scored across samples for each gene. **(E)** Immunomodulators median gene expression levels for D: GCA and L: IACSRCC group. Heatmap TPM (transcript per million) value is the median of all samples from respective groups, *z*-score normalized across the two groups.

**Figure 5:**
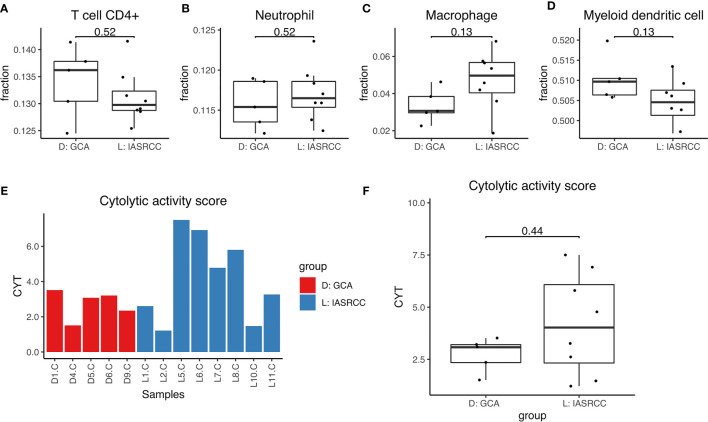
Immune cell infiltration and cytolytic activity of GCA *vs*. IACSRCCs. **(A–D)** Immune cell infiltration estimate comparison. Infiltration % of 6 types of immune cells was estimated for each sample with TIMER using gene expression data from RNA-seq. 2-sided Wilcoxon test was used. P-value < 0.05 was seen as statistically significant. **(E)** Cytolytic activity score for samples in D: GCA and L: IACSRCC groups. Cytolytic activity represents cytotoxic T cell activity and is calculated as the geometric mean of GZMA and PRF1 gene expression levels. **(F)** Cytolytic activity score comparison for D: GCA *vs*. L: IACSRCC group. 2-sided Wilcoxon test was used. P-value < 0.05 was seen as statistically significant.

First, we observed differences in the immune landscape between GCA (D group) and colorectal adenocarcinoma as well as IACSRCC (L group). Using gene expression data and TIMER immune cell infiltration estimation package, we predicted infiltration level of six types of immune cells critical to the tumor immune microenvironment. We found that GCA had a higher level of B-cell infiltration compared to colorectal adenocarcinoma (**Figure 3A**), but lower B-cell infiltration compared to IACSRCC with trend toward significance (**Figure 4B**), and significantly higher CD8+ T-cell infiltration levels compared to colorectal adenocarcinoma (**Figure 3E**), but lower CD8+ T-cell infiltration levels compared to IACSRCC (**Figure 4C**). We did not observe differences in levels of CD4+ T cells, neutrophils, macrophages, or myeloid dendritic cells between GCA and colorectal adenocarcinoma (**Figures 3B–D, F**) as well as between GCA and IACSRCC (**Figures 5A–D**). For all immune cell infiltration levels of all D: GCA and L: IACSRCC samples, see **Figure 4A**. We also compared levels of immunomodulation between GCA and IACSRCC using gene expression levels of 74 genes involved in immunomodulation. We found for inhibitory and stimulatory types of immunomodulators, GCA had a higher level of immunomodulation for some factors, while lower for other factors compared to IACSRCC. However, for various HLAs, we found GCA had lower expression levels for all HLA subtypes except HLA-B (**Figure 4E**). For immunomodulation status of individual D: GCA and L: IACSRCC samples, see **Figure 4D**. We also assessed the level of cytolytic T cell activity by geometric mean expression levels of GZMA and RPF1 and found no difference in cytolytic T-cell activity between GCA and IACSRCC (**Figure 5F**). For cytolytic T-cell activity levels of all D: GCA and L: IACSRCC samples, see **Figure 5E**.

### Specific Gene Expression, Biological Pathways, and Mutations Were Enriched in GCA Compared to Colorectal Adenocarcinoma and IACSRCC

**Figure 6:**
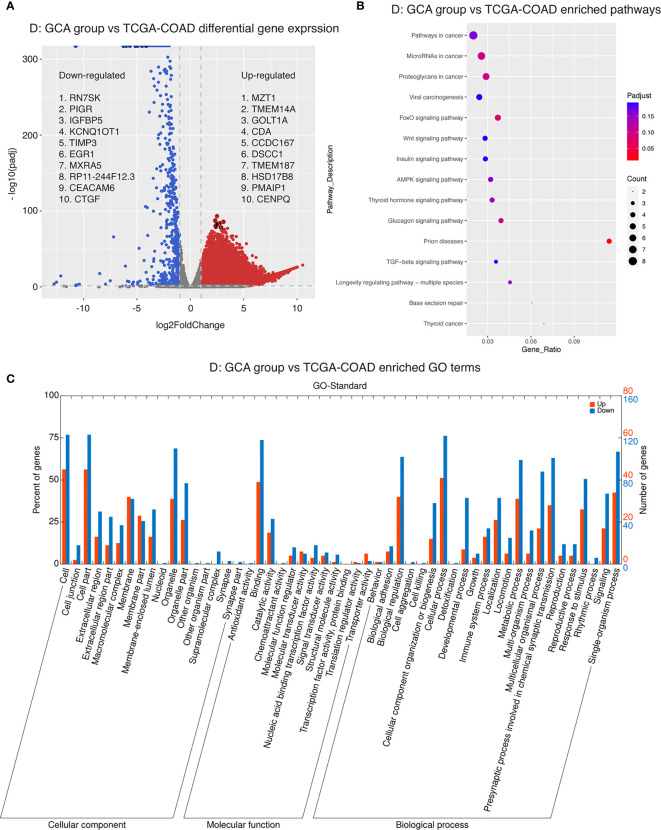
Transcriptomics landscape of GCA *vs*. colorectal adenocarcinoma. **(A)** Differential gene expression between GCA and colorectal adenocarcinoma. *p*-adjusted < 0.05 was considered significant. Genes upregulated in GCA with log2 fold change > 1 were colored in red. Genes downregulated in GCA with log2 fold change < −1 were colored in blue. **(B)** Pathway enrichment in GCA compared to colorectal adenocarcinoma. Five pathway databases (KEGG PATHWAY, PID, BioCyc, Reactome, and Panther) and five human disease databases (OMIM, KEGG DISEASE, FunDO, GAD, and NHGRI GWAS Catalog) were used to find pathways or disease in which differentially expressed gene set was overrepresented. **(C)** GO (gene ontology) term enrichment in GCA compared to colorectal adenocarcinoma. Gene ontology terms represent biological function categories while pathway analysis involves enrichment for metabolic and signaling pathways.

**Figure 7:**
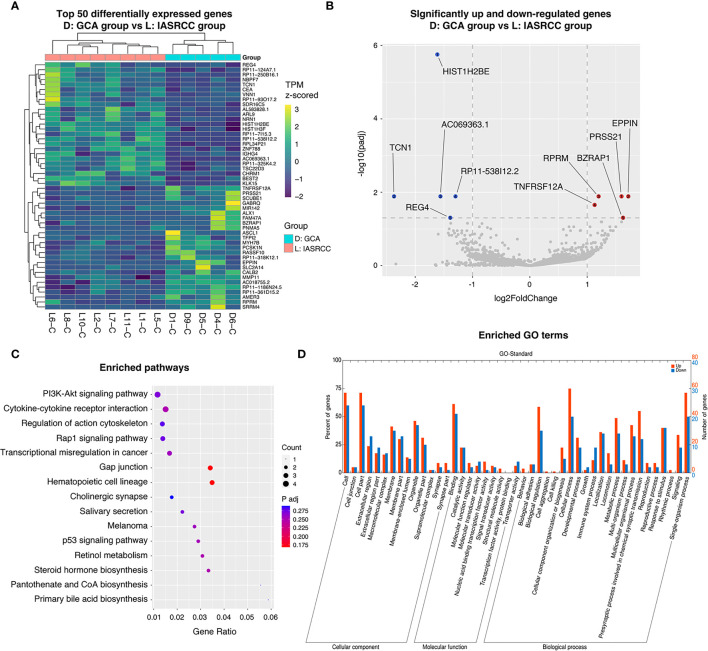
Transcriptomics landscape of GCA *vs*. IACSRCC. **(A)** Heatmap of top 50 differentially expressed genes between GCA and IACSRCC. Values represent TPM (transcript per million) *z*-scored across samples for each gene. Genes and samples were hierarchically clustered with dendrograms drawn on left and top of heatmap. **(B)** Differential gene expression between GCA and IACSRCC. *p*-adjusted < 0.05 was considered significant. Genes upregulated in GCA with log2 fold change > 1 were colored in red. Genes downregulated in GCA with log2 fold change < −1 were colored in blue. Top five up- and downregulated genes are labeled. **(C)** Pathway enrichment in GCA compared to IACSRCC. Five pathway databases (KEGG PATHWAY, PID, BioCyc, Reactome, and Panther) and five human disease databases (OMIM, KEGG DISEASE, FunDO, GAD, and NHGRI GWAS Catalog) were used to find pathways or disease in which differentially expressed gene set was overrepresented. **(D)** GO (gene ontology) term enrichment in GCA compared to IACSRCC. Gene ontology terms represent biological function categories while pathway analysis involves enrichment for metabolic and signaling pathways.

Next, we conducted differential gene expression analysis and GO and pathway enrichment analysis to see if specific genes are up- or downregulated in GCA compared to colorectal adenocarcinoma and IACSRCC, and if those genes were enriched in specific GO terms and metabolic, signaling, and disease pathways. We found that compared to colorectal adenocarcinomas, the top 10 upregulated genes in order of statistical significance were MZT1, TMEM14A, GOLT1A, CDA, CCDC167, DSCC1, TMEM187, HSD17B8, PMAIP1, and CENPQ (**Figure 6A**). Many downregulated genes had identically high statistical significance; therefore, the top 10 downregulated genes in order of effect size were RN7SK, PIGR, IGFBP5, KCNQ1OT1, TIMP3, EGR1, MXRA5, RP11-244F12.3, CEACAM6, and CTGF (**Figure 6A**). Pathway enrichment revealed enrichments in the following cancer-related pathways in the order of significance: FoxO signaling, proteoglycans, glucagon, thyroid hormone, AMPK, viral, and general cancer pathways (**Figure 6B**). In terms of enriched GO terms, the top five upregulated and downregulated GO terms all involve cell, cell part, binding, organelle, and cellular process (**Figure 6C**). Between GCA and IACSRCC, we found that these two groups can be well-separated using the top 50 differentially expressed genes, as evidenced by separately clustered GCA samples (salmon color) and IACSRCC samples (cyan color) (**Figure 7A**). Compared to the IACSRCC group, the top five upregulated genes in the GCA group were PRSS21, EPPIN, RPRM, TNFRSF12A, and BZRAP1. The top five downregulated genes were HIST1H2BE, TCN1, AC069363.1, RP11-538I12.2, and REG4 (**Figure 7B**). Pathway enrichment revealed enrichments in the following cancer-related pathways in the order of significance: hematopoietic cell lineage, Gap junction, cytokine–cytokine receptor interaction, steroid hormone biosynthesis, retinol metabolism, p53 signaling pathway, transcriptional mis-regulation in cancer, and PI3K-Akt signaling pathway (**Figure 7C**). For GO term enrichment, the top five upregulated and downregulated GO terms all involve cell, cell part, binding, organelle, and cellular process (**Figure 7D**). We also assessed gene mutation differences between GCA and IACSRCC. We observed that the two groups shared mutations in a number of genes (FAM47C, LOC101928841, FLG2, RP1L1, ZNF208, ZNF729, FLG, RPTN, CCDC168, and CDH23), albeit at different frequencies. However, the vast majority of the 30 most frequently mutated genes were different between the two groups (**Supplementary Figure S1**), further suggesting GCA is an unique genomic entity.

## Discussion

GCA is a very rare amphicrine tumor, previously considered to exist exclusively in the appendix. However, the extra-appendiceal cases suggested that GCA could occur outside the appendix. Most textbooks and literature listed GCA as an exclusive tumor in the appendix; therefore, most pathologists were unwilling to diagnose an extra-appendiceal GCA. Of the 12 GCAs in our study, there are only three appendiceal cases, which support our speculation. Some investigators used the term “amphicrine carcinoma” for the extra-appendiceal GCAs (5). Amphicrine carcinomas are characterized by both neuroendocrine and glandular differentiation occurring in the same cell (19). These tumors are also rare, with only scattered reports in the stomach (20), pancreas (21), lung (22), and liver (19). However, almost all amphicrine carcinomas reported lacked the classic low-grade GCA component (small round to oval tumor clusters or simple trabecular architecture composed of goblet cells) (19, 21, 23, 24). Therefore, GCA should be considered as one kind of amphicrine carcinoma with specific morphological characteristics. The classic low-grade component of GCA is the key point to make a correct diagnosis. In addition, before we diagnose a primary extra-appendiceal GCA, it is necessary to carefully differentiate these tumors from metastasis from primary appendiceal GCA.

GCA, especially in high-grade patients, had a poorer outcome than gastrointestinal low-grade NET (carcinoid). Vascular invasion and perineural invasion are very common, and five patients (41.7%) died of disease in 5 years in our data. The aggressive behavior supports reclassifying appendiceal goblet cell carcinoids as GCAs. Histologic grade correlated with overall survival independent of stage, so pathologists should provide an accurate grade for GCAs.

GCA usually shows immunoreactivity for neuroendocrine markers, such as chromogranin A, synaptophysin, and CD56 in variable numbers of tumor cells, but these stains are not required for diagnosis (1). Theoretically, GCAs lacking neuroendocrine immunoreactivity can exist. All 12 GCAs were positive for at least one of three neuroendocrine markers (including chromogranin A, synaptophysin, and CD56) in our patients. Chromogranin A was the most sensitive marker for GCA, positive in 10 (83.3%) GCAs in the present study. Unlike GCA, synaptophysin is more sensitive in the gastrointestinal NET (1). Colorectal NETs are usually positive for SSTR2 (1), but all GCAs were negative for SSTR2 in our study, which suggests that GCA should be classified into adenocarcinoma rather than carcinoid. Somatostatin analogs may be used in NETs when the SSTR status is positive (25). The negativity for SSTR2 indicates that somatostatin analogs may be ineffective for GCAs.

Morphologically, the large cohesive signet ring cell aggregates in IACSRCCs are very similar to the large aggregates or fused goblet cell clusters in the high-grade GCAs. The classic low-grade component of GCA is an essential character to differentiate GCA from IACSRCC. Most IACSRCCs were negative for neuroendocrine markers, and only one case showed focal stain for CD56 in the present 10 IACSRCCs. Although the neuroendocrine stains are not a requisite for the diagnosis of GCA, neuroendocrine immunoreactivity is still useful to differentiate GCA from IACSRCC.

GCA showed distinct tumor immune microenvironment compared to both colorectal adenocarcinoma as well as IACSRCC. GCA had differing infiltration levels of two crucial anti-tumor immune cells—B cells and CD8+ T cells, compared to colorectal adenocarcinoma as well as IACSRCC. B cell’s function in the tumor microenvironment is mainly anti-tumor. It produces tumor-reactive antibodies and primes CD4+ and CD8+ T cells (26). CD8+ T cells, especially the cytotoxic variety, are bona fide tumor-killing immune cells (27). It appears that GCA’s B- and CD8+ T-cell levels are between that of high immune cell-infiltrated IACSRCC and low immune cell-infiltrated colorectal adenocarcinoma. Tumor-infiltrating CD8+ T cells have been shown to be a beneficial prognostic factor in a number of cancers (28–30). CD8+ T-cell levels has even been shown to provide additional prognostic value beyond traditional TNM staging in colorectal cancer (31). Recent studies have also found that tumor-infiltrating B cells have a positive impact on survival across cancer types, and appears to also enhance the positive prognostic impact of CD8+ T cells (32). Assessing the prognostic impact of B-cell and CD8+ T-cell infiltration in GCA would be of great interest in future studies.

GCA showed distinct gene expression patterns compared to colorectal adenocarcinoma as well as the morphologically similar IACSRCC. Within the top 10 upregulated genes in GCA compared to colorectal adenocarcinoma, evidence suggests that a number of them are putative oncogenes. For example, TMEM14A, coding for a transmembrane protein, was shown to be abnormally expressed in various cancers (33). GOLT1A, a Golgi transport homolog, is overexpressed in breast cancer, and low expression is associated with good prognosis (34). DSCC1, involved in DNA replication and sister chromatid cohesion, is actually frequently upregulated in colorectal cancer cells (35), where it is shown here to be even more upregulated in GCA compared to colorectal adenocarcinoma. HSD17B8, a steroid dehydrogenase, plays a crucial role in the development of endocrine and endocrine-related cancers (36) and was also found to be upregulated in GCA compared to colorectal adenocarcinoma, which may reflect the endocrine nature of GCA. Most interestingly, numerous genes whose expressions are unique to colorectal adenocarcinoma were downregulated in GCA. For example, KCNQ1OT1, a long non-coding RNA, acts as an oncogene in colorectal cancer through the PI3K/AKT pathway (37), but was found here to be downregulated in GCA. MXRA5, a matrix-remodeling protein, was identified as a colorectal cancer biomarker (38) and was also found to be downregulated in GCA. Compared to IACSRCC, GCA was upregulated in PRSS21, EPPIN, RPRM, TNFRSF12A, and BZRAP1, and downregulated in HIST1H2BE, TCN1, AC069363.1, RP11-538I12.2, and REG4. Many of these genes are also cancer-related. For example, RPRM is a gastric cancer biomarker (39), and TCN1 high expression was linked to negative colon cancer prognosis (40). Distinct gene expression signature of GCA involving cancer-related genes may not only aid in the differential diagnosis of GCA, but also pave the way for a deeper understanding of the molecular oncogenic pathways involved in GCA.

In summary, this study demonstrated that GCA can be seen as a distinct entity, with unique immune and molecular features compared to both colorectal adenocarcinoma as well as IACSRCC. The number of extra-appendiceal GCA was underestimated in Chinese patients. The classic low-grade component of GCA and the immunoreactivity for neuroendocrine markers are key points to diagnosing GCA.

## Data Availability Statement

The datasets presented in this article are not readily available because dataset is used in other ongoing studies. Requests to access the datasets should be directed to the corresponding author X-MX at xiaoming.xing@qdu.edu.cn.

## Ethics Statement

The studies involving human participants were reviewed and approved by the Ethics Committee of the Affiliated Hospital of Qingdao University. The patients/participants provided their written informed consent to participate in this study.

## Author Contributions

D-LL, L-LW, PZ, W-WR, WW, and L-XZ contributed to conception, design of the study, formal analysis, and manuscript writing. MH and HB contributed to statistical analysis, visualization, and manuscript writing. HB, KL, XW, YS, and X-MX designed, supervised, and provided resources for this study. All authors contributed to manuscript revision, read, and approved the submitted version.

## Funding

This work was supported by the National Natural Science Foundation of China Grants (No. 81972329).

## Conflict of Interest

Authors MH, HB, KL, XW, and YS are employed by Nanjing Geneseeq Technology Inc.

The remaining authors declare that the research was conducted in the absence of any commercial or financial relationships that could be construed as a potential conflict of interest.

## Publisher’s Note

All claims expressed in this article are solely those of the authors and do not necessarily represent those of their affiliated organizations, or those of the publisher, the editors and the reviewers. Any product that may be evaluated in this article, or claim that may be made by its manufacturer, is not guaranteed or endorsed by the publisher.
