# Supplementary figures and images for: Gastrointestinal Goblet Cell Adenocarcinomas Harbor Distinctive Clinicopathological, Immune, and Genomic Landscape
Source: Front Oncol. 2021 Nov 5;11:758643. doi: 10.3389/fonc.2021.758643 (PMC8603204; doi:10.3389/fonc.2021.758643)

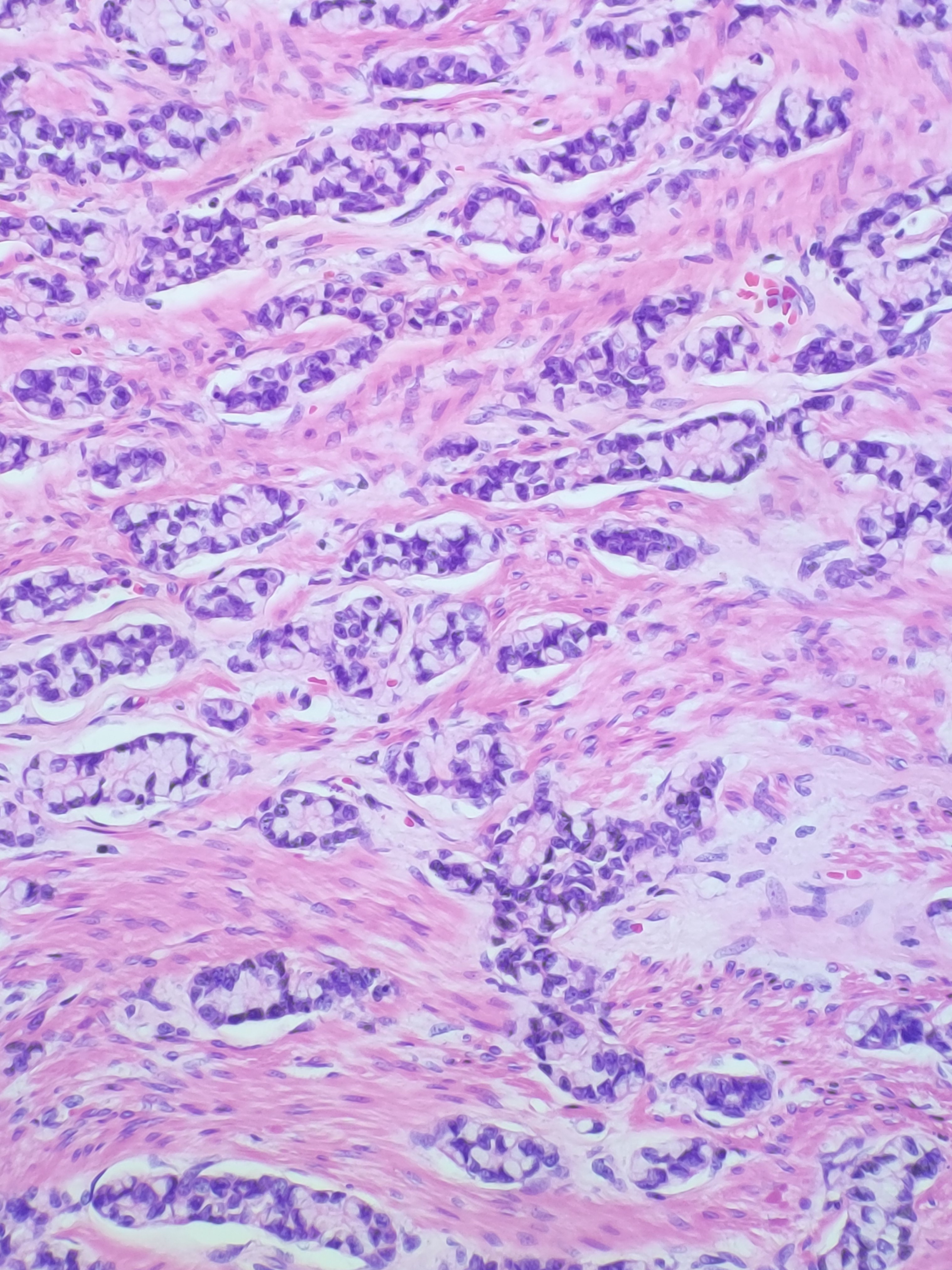

Supplement: Supplementary file 1 [file DataSheet_1.zip › Figure 1A_hi_res.JPEG]

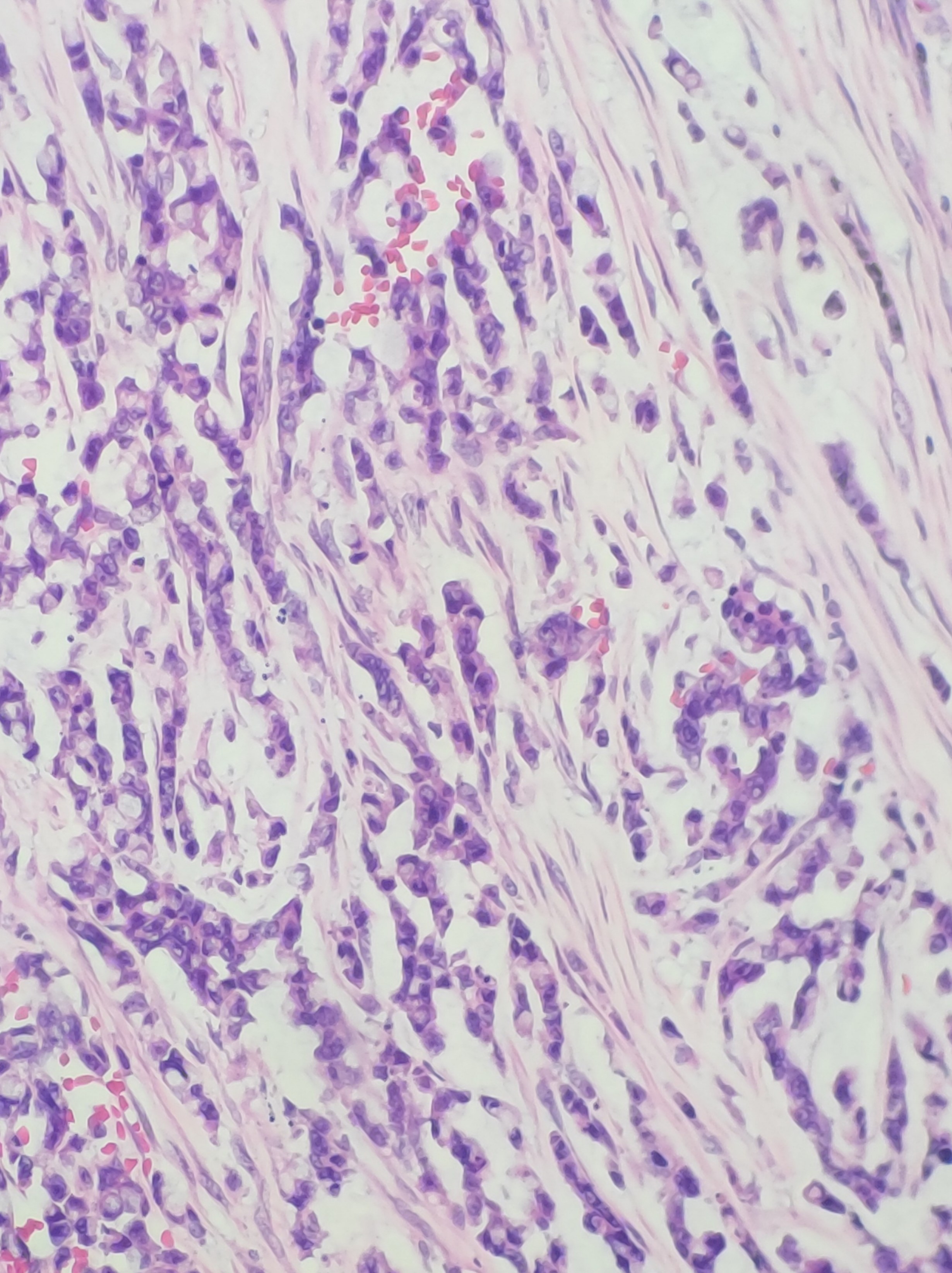

Supplement: Supplementary file 1 [file DataSheet_1.zip › Figure 1B_hi_res.JPEG]

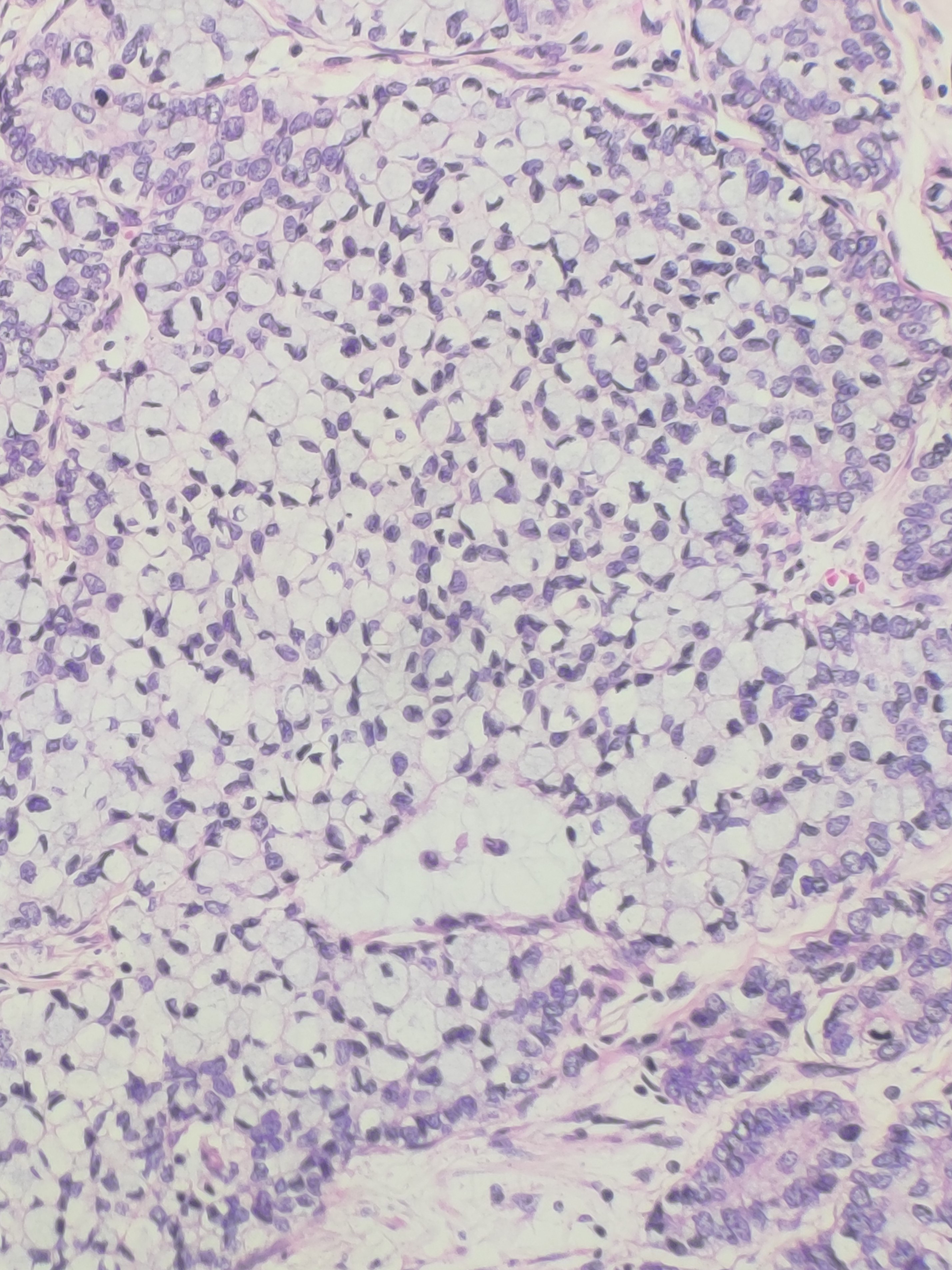

Supplement: Supplementary file 1 [file DataSheet_1.zip › Figure 1C_hi_res.JPEG]

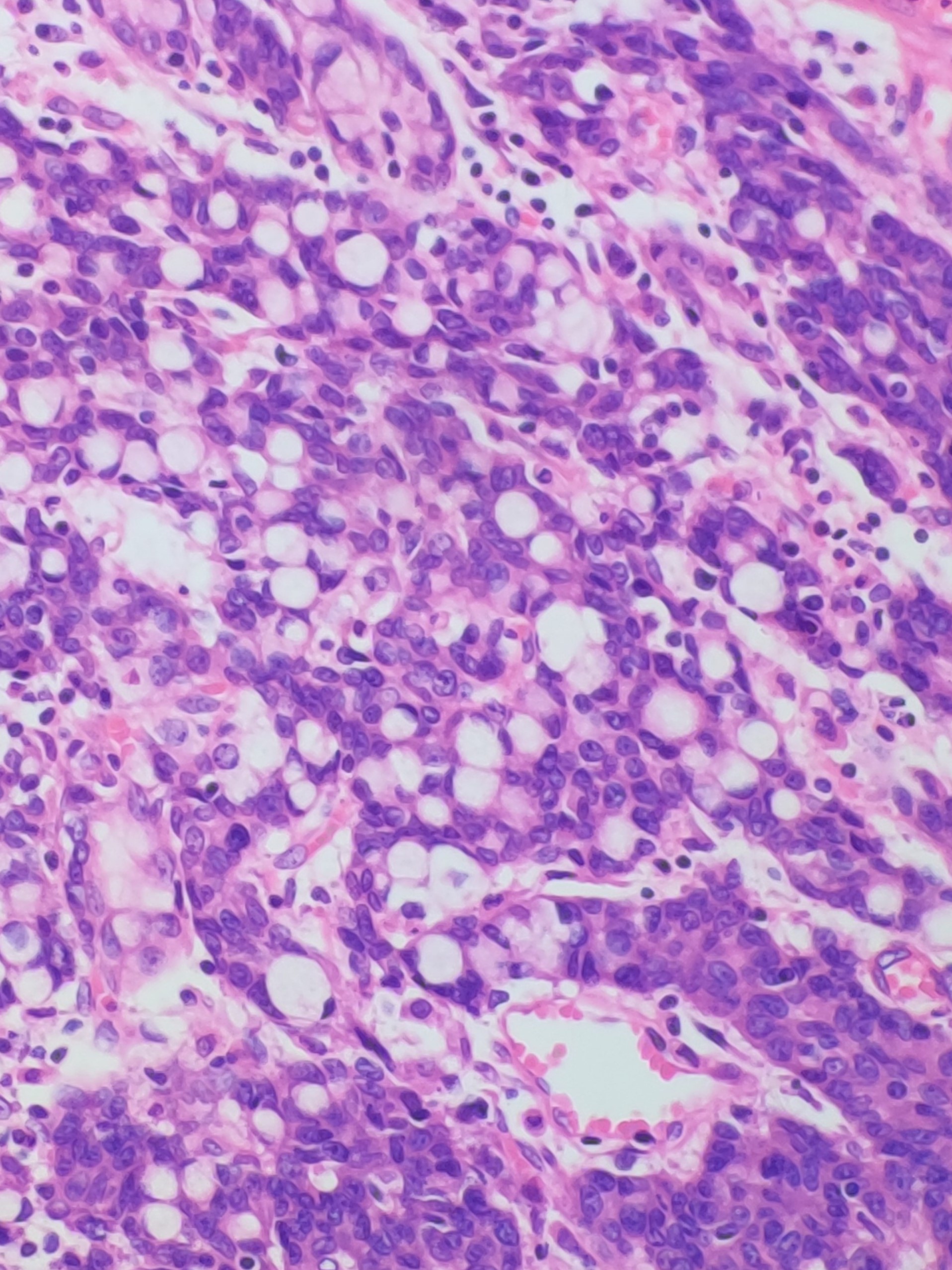

Supplement: Supplementary file 1 [file DataSheet_1.zip › Figure 1D_hi_res.JPEG]

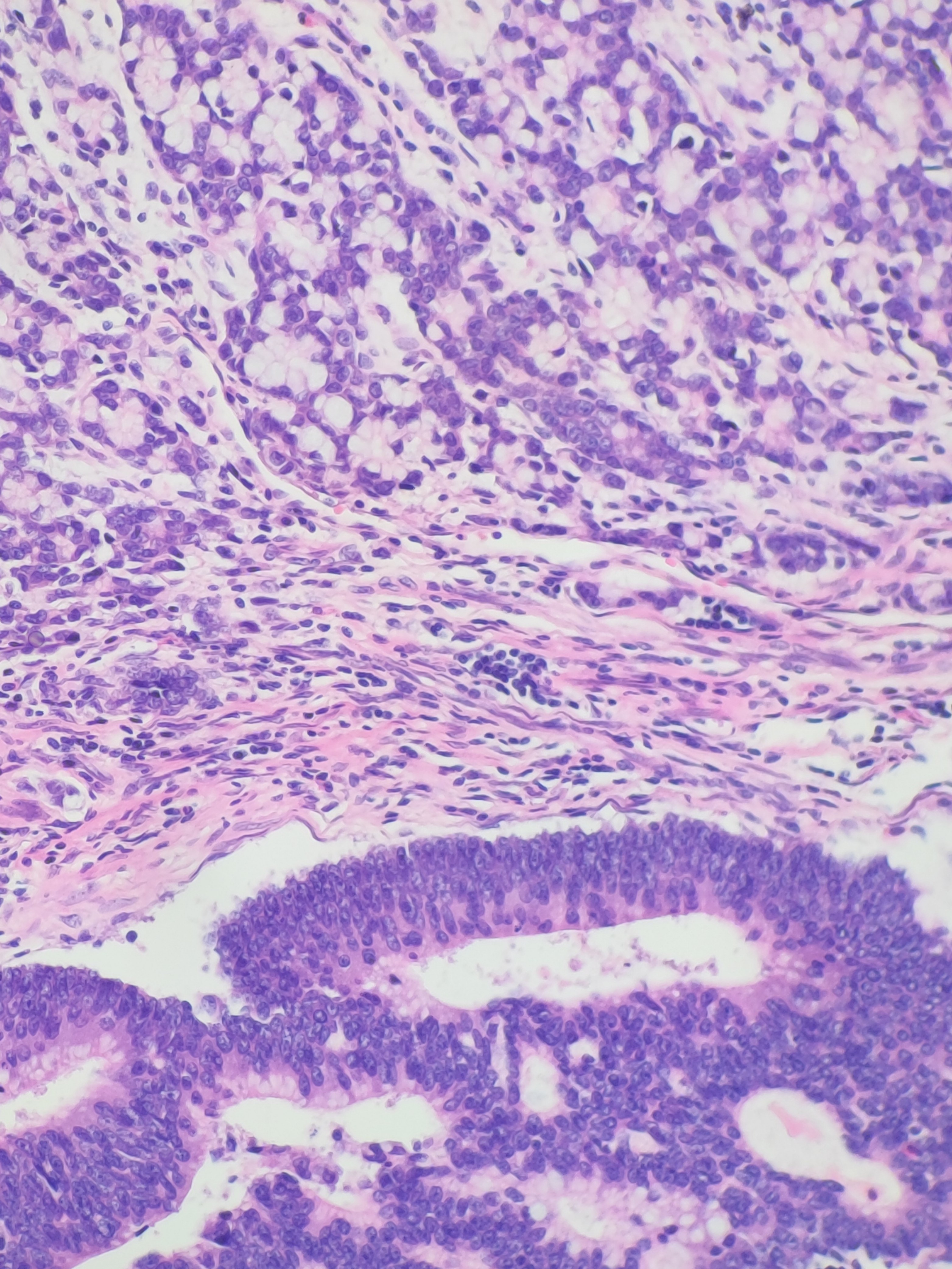

Supplement: Supplementary file 1 [file DataSheet_1.zip › Figure 1E_hi_res.JPEG]

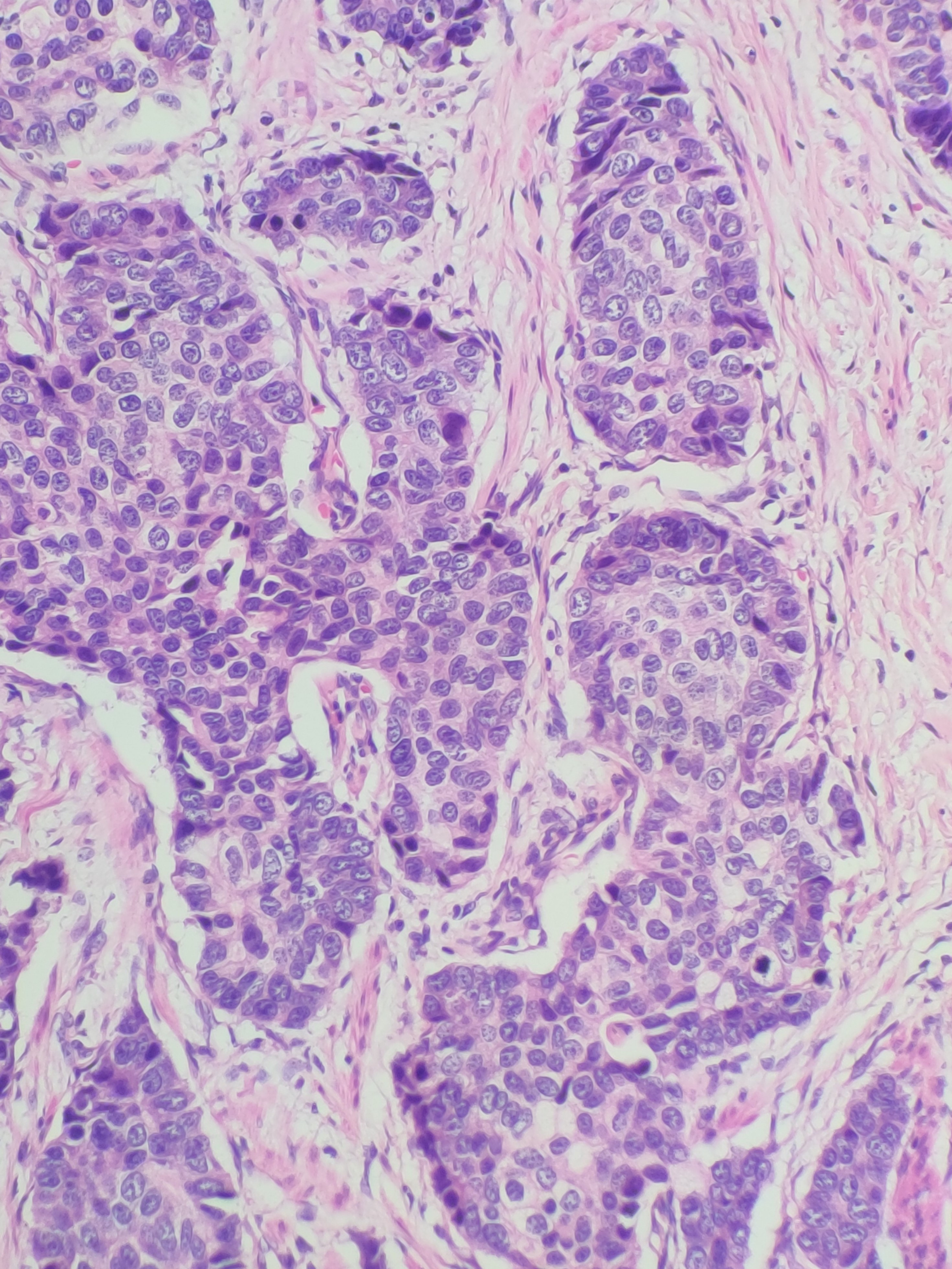

Supplement: Supplementary file 1 [file DataSheet_1.zip › Figure 1F_hi_res.JPEG]

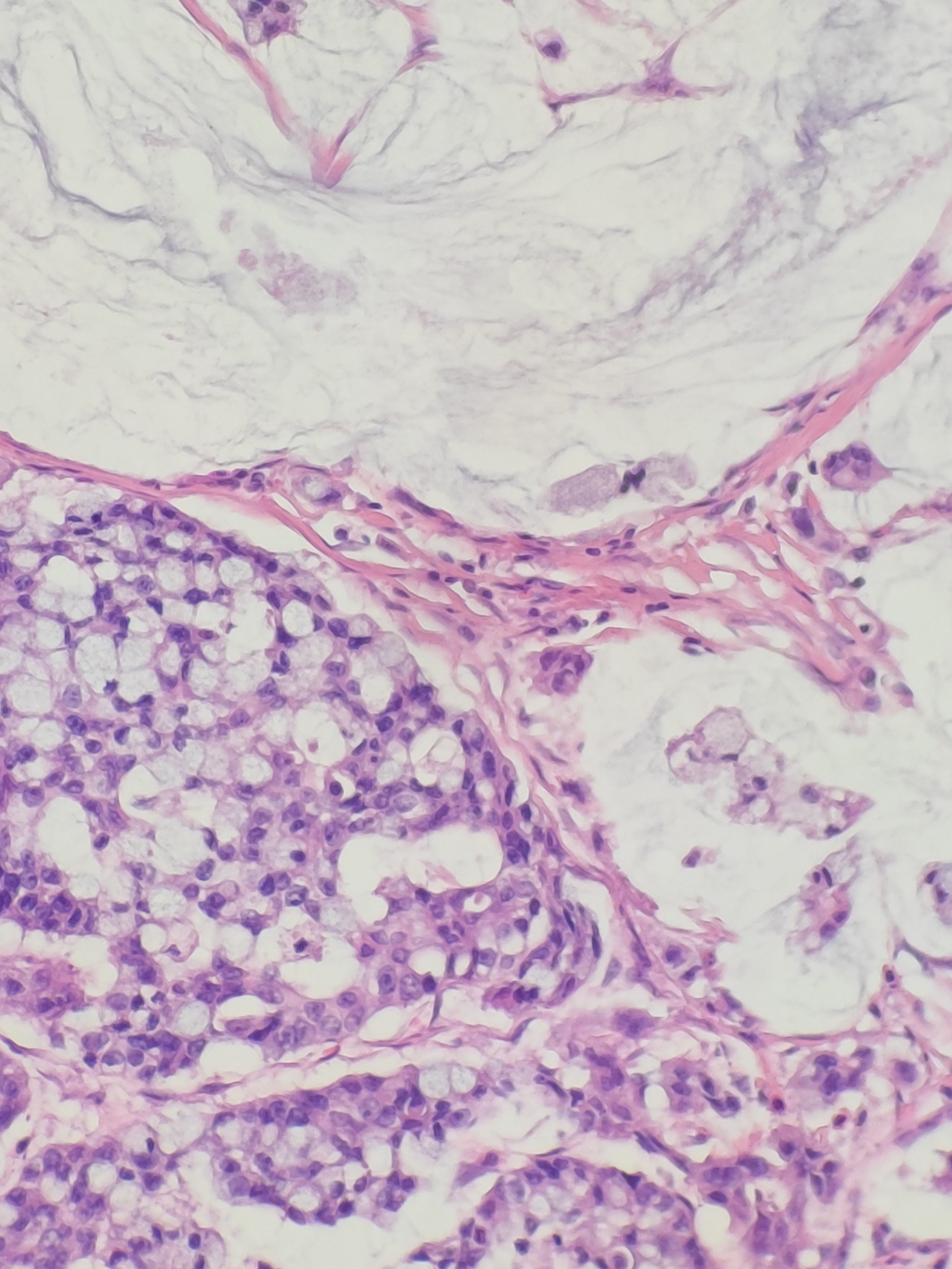

Supplement: Supplementary file 1 [file DataSheet_1.zip › Figure 1G_hi_res.JPEG]

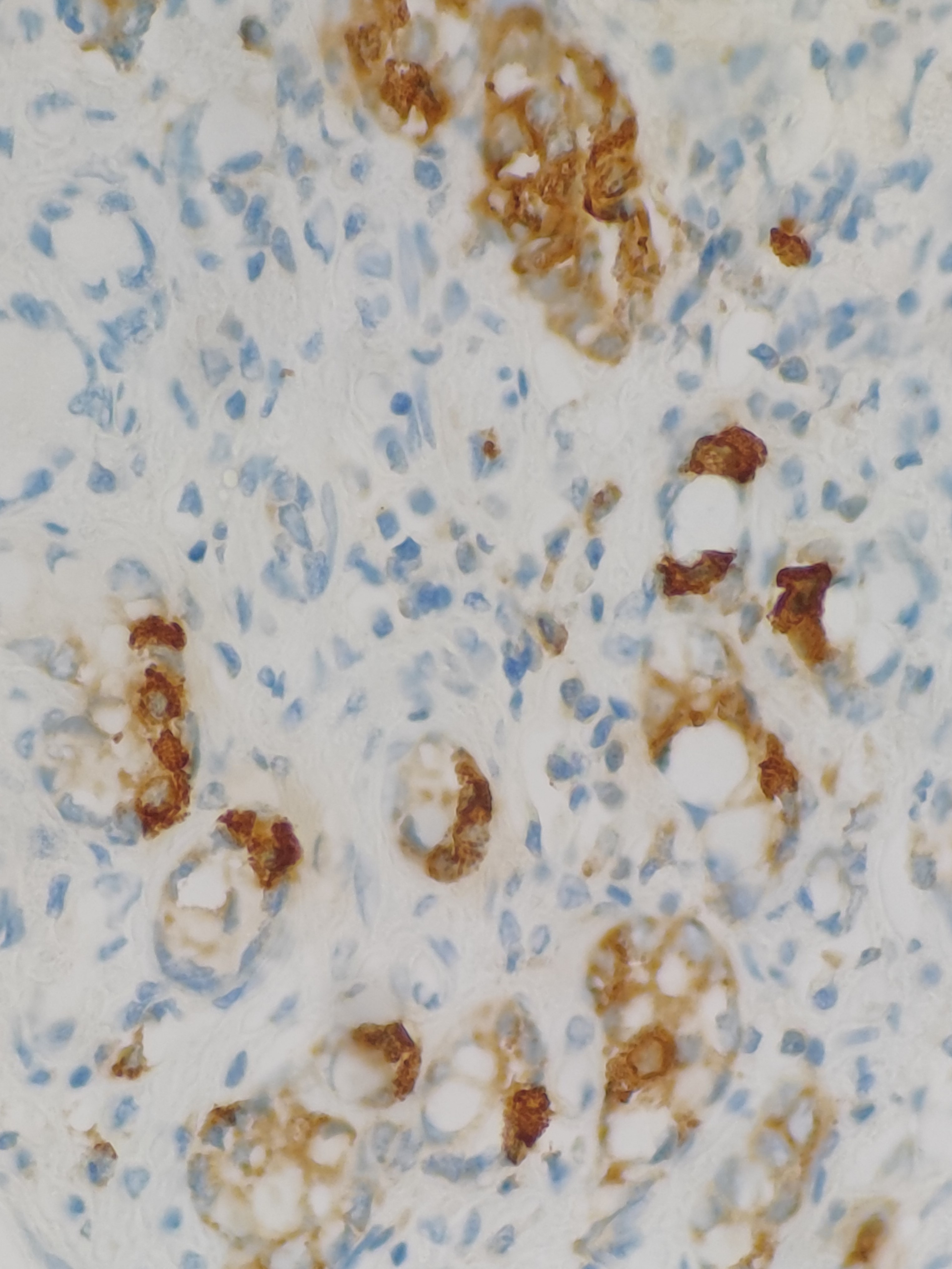

Supplement: Supplementary file 1 [file DataSheet_1.zip › Figure 1H_hi_res.JPEG]

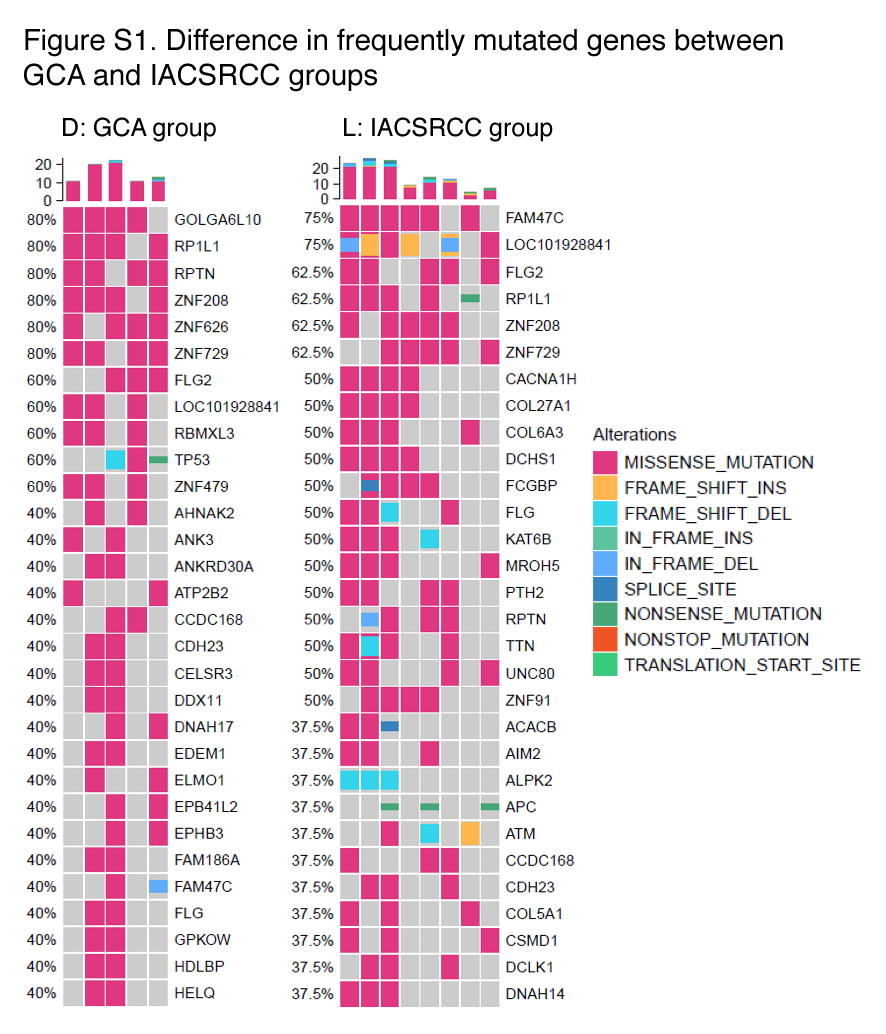

Supplement: Supplementary file 2 [file Image_1.tif]
