# Supplementary material for: Gastrointestinal Goblet Cell Adenocarcinomas Harbor Distinctive Clinicopathological, Immune, and Genomic Landscape
Source: Front Oncol. 2021 Nov 5;11:758643. doi: 10.3389/fonc.2021.758643 (PMC8603204; doi:10.3389/fonc.2021.758643)
Supplement: Supplementary file 3 [file Table_1.docx]

**Table S1**. Clinicopathologic characteristics of IACSRCCs.

|  |  |  | |  | Histologic Findings | | | | |  |  | |
| --- | --- | --- | --- | --- | --- | --- | --- | --- | --- | --- | --- | --- |
| Case No. | Age(years)/Sex | | Location | | Size (cm) | Predominant component | Vascular invasion | Perineural invasion | TNM | | | Follow-up (months) |
| 1 | 73/F | | Transverse colon | | 7 | Signet ring cell carcinoma | - | + | T3N0M0 | | | NET (55) |
| 2 | 58/F | | Ascending colon | | 10 | Mucinous carcinoma | + | - | T3N1M0 | | | NET (54) |
| 3 | 61/M | | Rectum | | 4 | Signet ring cell carcinoma | + | + | T3N2bM0 | | | NET (51) |
| 4 | 37/M | | Ascending colon | | 11 | Signet ring cell carcinoma | - | - | T3N1M0 | | | NET (47) |
| 5 | 80/M | | Rectum | | 6 | Signet ring cell carcinoma | - | + | T3N2bM0 | | | DOD (5) |
| 6 | 42/M | | Ascending colon | | 5 | Signet ring cell carcinoma | + | + | T3N2bM0 | | | DOD (8) |
| 7 | 57/M | | Descending colon | | 7 | Signet ring cell carcinoma | + | + | T3N0M0 | | | NET (19) |
| 8 | 58/M | | Rectum | | 6 | Signet ring cell carcinoma | + | + | T3N2bM0 | | | NET (40) |
| 9 | 68/M | | Rectum | | 4 | Signet ring cell carcinoma | - | + | T3N1bM0 | | | NET (37) |
| 10 | 70/M | | Sigmoid colon | | 10.5 | Mucinous carcinoma | + | + | T3N2bM0 | | | DOD (20) |

F, female; M, male; NET, no evidence of tumor; DOT, died of disease.
