# Supplementary material for: Gastrointestinal Goblet Cell Adenocarcinomas Harbor Distinctive Clinicopathological, Immune, and Genomic Landscape
Source: Front Oncol. 2021 Nov 5;11:758643. doi: 10.3389/fonc.2021.758643 (PMC8603204; doi:10.3389/fonc.2021.758643)
Supplement: Supplementary file 4 [file Table_2.docx]

**Table S2**. Detailed immunohistochemical findings of GCA.

| Marker Case No. | Synaptophysin  (Proportion) | Chromogranin A  (Proportion) | CD56  (Proportion) | SSTR2 | Ki67 index |
| --- | --- | --- | --- | --- | --- |
| 1 | - | + (10%) | + (20%) | - | 70% |
| 2 | - | + (15%) | - | - | 55% |
| 3 | + (35%) | + (25%) | + (10%) | - | 55% |
| 4 | - | - | + (20%) | - | 45% |
| 5 | - | + (10%) | - | - | 25% |
| 6 | - | + (20%) | - | - | 45% |
| 7 | + (40%) | - | - | - | 30% |
| 8 | + (30%) | + (30%) | - | - | 50% |
| 9 | - | + (20%) | + (30%) | - | 50% |
| 10 | - | + (35%) | - | - | 25% |
| 11 | + (20%) | + (40%) | - | - | 40% |
| 12 | - | + (30%) | - | - | 10% |
